# Local adaptation of a bacterium is as important as its presence in structuring a natural microbial community

**DOI:** 10.1038/ncomms12453

**Published:** 2016-08-09

**Authors:** Pedro Gómez, Steve Paterson, Luc De Meester, Xuan Liu, Luca Lenzi, M. D. Sharma, Kerensa McElroy, Angus Buckling

**Affiliations:** 1 ESI and CEC, Biosciences, University of Exeter, Penryn Campus, Cornwall TR10 9FE, UK; 2 CEBAS-CSIC, Campus Espinardo, 30100 Murcia, Spain; 3 Institute of Integrative Biology, University of Liverpool, Liverpool L69 7ZB, UK; 4 Laboratory of Aquatic Ecology, Evolution and Conservation, KU Leuven, 3000 Leuven, Belgium; 5 CEC, Biosciences, University of Exeter, Penryn Campus, Cornwall TR10 9FE, UK; 6 Common wealth Scientific and Industrial Research Organisation (CSIRO), Canberra GPO Box 1700, Australia

## Abstract

Local adaptation of a species can affect community composition, yet the importance of local adaptation compared with species presence *per se* is unknown. Here we determine how a compost bacterial community exposed to elevated temperature changes over 2 months as a result of the presence of a focal bacterium, *Pseudomonas fluorescens* SBW25, that had been pre-adapted or not to the compost for 48 days. The effect of local adaptation on community composition is as great as the effect of species presence *per se*, with these results robust to the presence of an additional strong selection pressure: an SBW25-specific virus. These findings suggest that evolution occurring over ecological time scales can be a key driver of the structure of natural microbial communities, particularly in situations where some species have an evolutionary head start following large perturbations, such as exposure to antibiotics or crop planting and harvesting.

## Abstract (web summary)

Though both the presence and traits of a species can influence the dynamics of its ecological community, the effects of these factors are difficult to disentangle. Here, Gómez *et al*. demonstrate in a microbial mesocosm that local adaptation of a focal species can influence the community as much as the presence of the focal species *per se*.

## Graphical abstract

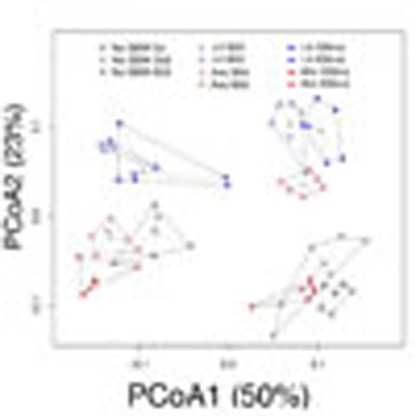

Community structure changes in response to environmental perturbation because some species perform better under the novel conditions than others^1^. In parallel with recent theory^2^, there is a growing body of empirical work that suggests contemporaneous evolution (evolution occurring on the same time scale as ecological changes) may contribute to these changes in community composition^3^,^4^,^5^,^6^,^7^. Studies using simplified laboratory communities of microbial organisms^8^,^9^,^10^,^11^ show that contemporaneous evolution can affect interactions within- and between-trophic levels, and historical effects of microevolution have been shown to affect subsequent community structure in more complex settings^12^,^13^,^14^,^15^.

A number of studies have attempted to determine the relative impact of contemporaneous evolution versus ecological variables on community structure^7^,^14^,^16^. For example, local adaptation of *Daphnia magna* was found to be as important as the presence of predatory fish in driving the structure of zooplankton communities in mesocosms^7^. However, while this work provides important information on the relative importance of specific environmental variables and adaptation to these variables, a more general way to estimate importance of local adaptation is to measure its effect relative to the presence of the evolving species *per se*.

Here, we concurrently determine how both the presence of a focal species and local adaptation of this species affect the structure of a natural soil (compost) microbial community, and consequently the relative importance of contemporaneous evolution to species presence *per se*. As a focal species, we use the bacterium *Pseudomonas fluorescens* SBW25 (ref. 17), which we have previously shown to be able to adapt rapidly to a sterile compost environment by diversifying into resource niche specialists^18^. We estimate the ecological impact of *P. fluorescens* SBW25 by comparing how its presence versus absence affects the natural community structure in compost microcosms propagated over 60 days. Likewise, we estimate the effect of contemporaneous evolution by comparing how SBW25 that had been adapted to the compost microcosms (for 48 days) and ancestral SBW25 affect community structure. To increase the potential importance of local adaptation, we propagated microcosms at an elevated temperature (26 °C), representing a novel environment for both the compost-dwelling community (novel temperature) and sugar beet-associated SBW25 (ref. 17; novel temperature and substrate). Finally, we explore the robustness of our findings by determining how an additional ecologically relevant selection pressure may affect the importance of species presence and additional local adaption affects community structure. We have previously shown that a highly SBW25-specific bacteriophage, SBW25φ2 (ref. 19), impose strong selection on SBW25 in soil^20^,^21^, as apparent from both density reductions and resistance evolution, hence we inoculated this virus into additional microcosms containing ancestral and locally adapted SBW25.

We found that contemporaneous adaptive evolution of a single species can play as great a role as species presence in structuring natural microbial communities. Crucially, we detected large differences in community composition between treatments, with the effect of adaptation as great as the effect of the presence of SBW25 *per se*. These results provide an experimental verification of evolutionary priority effects theory in natural microbial communities, with a particular relevance for understanding how natural microbial communities become established, and hence the impact they may have on ecosystem function and host health.

## Results

### Local adaptation of *Pseudomonas fluorescens* SBW25 to compost

**Figure 1:**
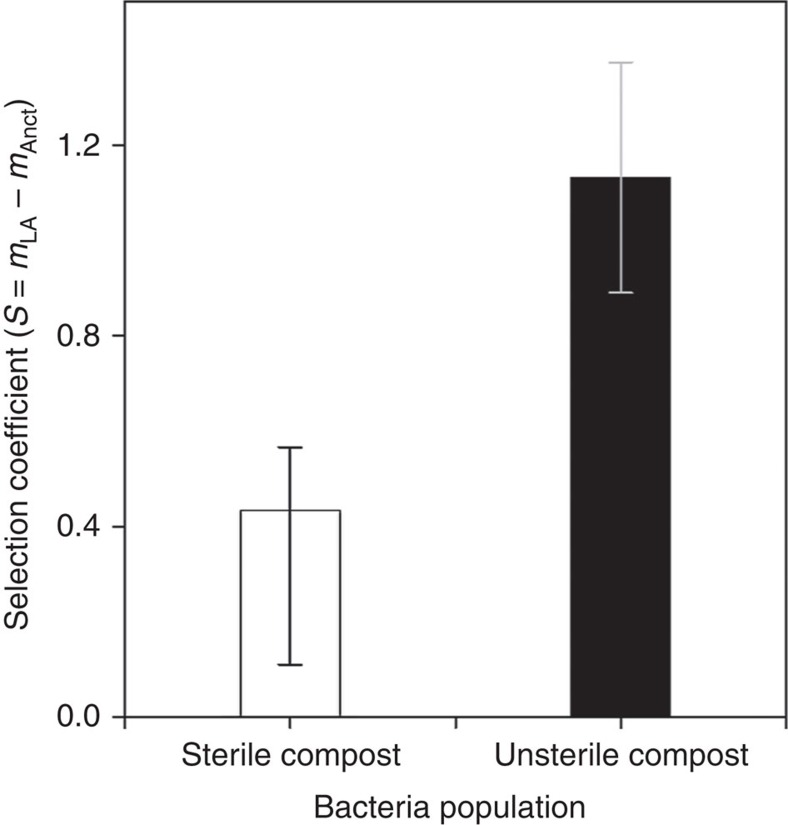
Fitness of locally adapted SBW25. Mean (±s.e.m.) selection coefficient of evolved SBW25 (after 48 days cultivation in sterile compost at 26 °C) determined by competing against its ancestor for 5 days in both sterile and unsterile compost. Note that selection coefficient (*S*) was calculated here by the difference between the estimated Malthusian parameter of the locally adapted SBW25 population (*m*_LA_) and the ancestral SBW25-*lacZ* strain (*m*_Anct_), where a value of zero indicates equal fitness.

We first determined whether the six SBW25 populations that had been previously cultured in sterile compost under equivalent abiotic conditions as used here^18^ had adapted to the compost environment. To this end, the relative fitness of SBW25 populations was measured by competing them against their *lacZ*-marked ancestor, after confirming no difference in competitive fitness of the unmarked and marked ancestor (one sample *t*-test; *n*=6; *t*=1.4 *P*>0.6). Large increases in competitive fitness relative to the ancestor were detected when measured in both the presence and absence of the natural communities (one sample *t*-test; *t*=0.03 *P*<0.01 in both cases, Fig. 1). To confirm that these phenotypic changes were correlated with genetic changes, we sequenced a single clone from each of the six populations. Non-synonymous mutations were detected in all populations (a list of either single-nucleotide polymorphisms or small insertions and deletions can be found as Supplementary Data 1). However, given that most mutated genes only occurred in single clones, and the few parallel changes that did occur affected genes of unknown functions, it is difficult to infer links between genomic and phenotypic changes. Unambiguous parallel changes occurred in the PFLU1093 and PFLU4198 genes, which were disrupted by insertions that led to frame-shifts. These genes encode a putative fimbrial usher protein and a putative sensory box GGDEF/EAL domain-containing protein that, in particular, are associated with the biogenesis of pili and the phosphorylation catalysis in response to detection of a chemical ligand or change in environment that initiate a change in cell activity, respectively.

### Changes in the population densities

**Figure 2:**
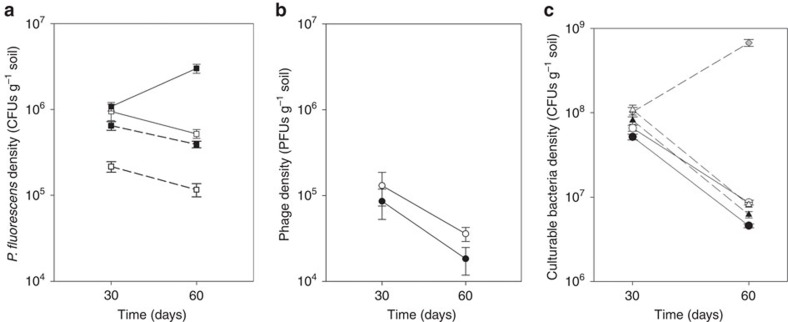
Bacterial population densities in the natural microbial community. (**a**) Mean densities (±s.e.m.) of the six replicates of ancestral (white square) and locally adapted (black square) *P. fluorescens* SBW25 (CFUs g^−1^ soil) in the presence of the natural microbial community (solid line) and the presence of phages (dash line). (**b**) Mean densities (±s.e.m.) of phage SBW25φ2 (plaque-forming units (PFUs)  g^−1^ soil) populations. (**c**) Mean densities (±s.e.m.) of the six replicates of the culturable bacteria (CFUs g^−1^ soil) in the natural microbial community with no SBW25 (grey diamond), ancestral SBW25 (white circle, white triangle) and locally adapted SBW25 (black circle, black triangle) in the absence (solid line) and presence (dashed line) of phages.

We determined the population densities of SBW25, SBW25φ2 and members of the natural microbial community that could be cultured *in vitro* under similar conditions to SBW25 bacterial communities after 30 and 60 days, ensuring ecological changes were measured over a comparable time scale to the 48 days of SBW25 evolution. Locally adapted SBW25 reached on average approximately three times greater mean population density than ancestral SBW25 in the presence of the natural community (Fig. 2a, Linear Mixed Effects Models (LMM), *F*_1,20_=82.2, *P*<0.001), providing a clear mechanism by which local adaptation might affect community composition. Phages reduced the density of the SBW25 approximately 4-fold (LMM*, F*_1,20_=123.5, *P*<0.001), with no differential reduction between ancestral and locally adapted SBW25 (Fig. 2a, SBW25 treatment by phage: *F*_1,20_=0.75, *P*=0.4). Phage densities were not significantly different when co-evolving with ancestral or locally adapted SBW25 (Fig. 2b, LMM, *F*_1,10_=3.8, *P*=0.08). Note that phage densities significantly decreased from day 30 to 60 (Fig. 2b, LMM, *F*_1,11_=30.3, *P*<0.001), while SBW25 densities typically decreased between these time points, except locally adapted SBW25 in the absence of phages increased (Fig. 2a, LMM, time by phage by SBW25 treatment: *F*_1,20_=5.5, *P*=0.3).

The density of culturable bacteria that grew under the same culture media on which SBW25 thrives was reduced approximately 10-fold by the presence of SBW25 in the absence of phages (Fig. 2c; LMM, *F*_2,15_=682, *P*<0.001). There was a further approximately 50% reduction resulting from the presence of locally adapted SBW25 (Fig. 2c; LMM, *F*_1,20_=25.88, *P*<0.001), while the presence of phages increased the density of culturable bacteria by approximately 50% (Fig. 2c; LMM, *F*_1,20_=16.88, *P*<0.001), with no interaction between phages and whether SBW25 had been locally adapted or not (Fig. 2c, LMM, *F*_1,20_=1.26, *P*=0.27). Densities of culturable bacteria decreased through time in the presence of SBW25, but increased in the absence of phages (Fig. 2c, LMM, time by SBW25 treatment: *F*_1,27_=326, *P*<0.001). In summary, these data show that while locally adapting SBW25 had a significant effect on the density of culturable bacteria, this effect size was marginal (approximately 5%) compared with that of the presence of SBW25 *per se*.

### Changes in the bacterial community structure

**Figure 3:**
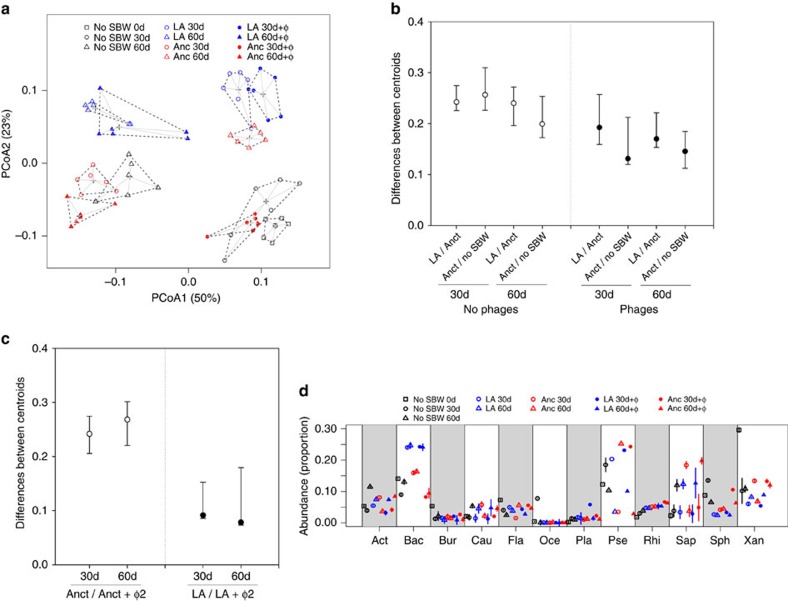
Changes in community composition. (**a**) Principal Coordinate Analysis (PCoA) plot of communities based on UniFrac distances. The percentage of variation explained is shown on each axis (calculated from the relevant eigenvalues). Replicates within treatments and time points have similar community compositions, while there are notable differences between treatments and time points. (**b**) Points show mean (95%±confidence intervals (CIs)) distances between treatment centroids at 30 and 60 days in the absence (open circles) and presence (closed circles) of phages. These centroids are the mean position of all the points in all coordinate directions from the PCoA plot, and their differences reflect the effect of adaptation and species presence on community change. The difference between ancestral and locally adapted treatments shows the magnitude of the effect of adaptation, while difference between presence and absence of SBW25 shows the magnitude of effect of species presence. (**c**) Points show mean (95%±CIs) distances between treatment centroids to show how phages (+φ2) affect community structure in the presence of either ancestral or evolved SBW25. (**d**) Common bacterial orders across treatments (>5% in at least one treatment–time point combination). Note consistently high frequency of *Bacillales* (Bac) across time in evolved SBW25 treatment. Act, *Actinomycetales*; Bur, *Burkholderiales*; Cau, *Caulobacterales*; Fla, *Flavobacteriales*; Oce, *Oceanospirillales*; Pse, *Pseudomonadales*; Rhi, *Rhizobiales*; Sap, *Saprospirales*; Sph, *Sphingobacteriales*; Xan, *Xanthomonadales*. Square represents day 0, circle represents day 30, triangle represents day 60; black represents No SBW25; blue represents locally adapted (LA), red represents ancestral (Anct); open symbols indicate no phage, closed symbols indicate with phage (+φ).

**Figure 4:**
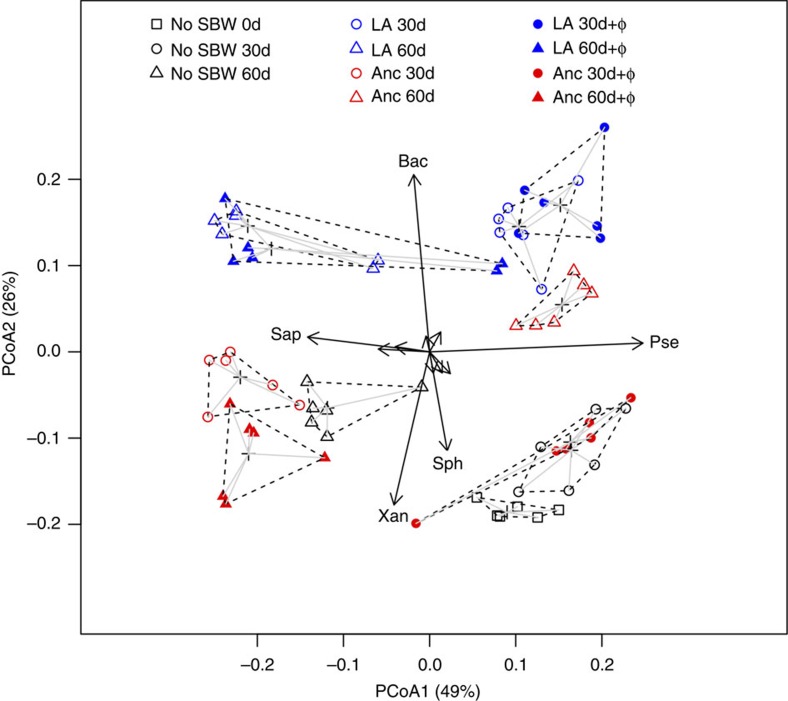
Differences in community composition. Principal Coordinate Analysis (PCoA) based on Bray–Curtis dissimilarity of bacterial orders between communities. PCA loadings for orders playing key roles in determining community differences are shown for comparison as arrows: Bac, *Bacillales*; Pse, *Pseudomonadales*; Sap, *Saprospirales*; Sph, *Sphingobacteriales*; Xan, *Xanthomonadales*. Square represents day 0, circle represents day 30, triangle represents day 60; black represents No SBW25; blue represents locally adapted (LA), red represents ancestral (Anc); open symbols indicate no phage, closed symbols indicate with phage (φ2).

Using a simple culture-independent method (amplicon sequencing of a variable region of 16S rDNA^22^) we detected high levels of diversity within each of the bacterial communities, with approximately 10^3^ distinct operational taxonomic units (OTUs) per community. Despite this high level of diversity, we detected large differences in the relative frequency of different OTUs between treatments (no phages: no SBW25, ancestral and locally adapted; phages: ancestral and locally adapted SBW25) and time points (Figs 3a and 4; all treatments and time points differed; PERMANOVA *P*<0.01 for all pairwise comparisons). Regardless of the presence of phages, differences in community composition (based on weighted UniFrac distances) between the locally adapted and ancestral SBW25 treatments were of a similar magnitude to those between the ancestral and no SBW25 treatments (Fig. 3a,b). There was a tendency for phages to reduce the impact of both SBW25 treatments on community structure (Fig. 3b), but phages had a much greater impact on community structure-imposed changes caused by ancestral than locally adapted SBW25 (Fig. 3c).

Although there were many differences in the frequency of specific groups of bacteria between treatments within time points, many of these differences were reversed between time points (Fig. 3d), hence removing any net effects between treatments. The notable exception was the *Bacillales* order, a common spore-forming group of soil bacteria, which had elevated frequencies in the presence of locally adapted SBW25 in both the presence and absence of phages, (Fig; two-sample *t*-test, *P* <0.001 for all comparisons). Note that the frequency of the order (or any lower taxonomic unit) to which SBW25 belongs, the *Pseudomonadales*, was not consistently elevated following the addition of ancestral or locally adapted SBW25, demonstrating that differences in community composition were not driven by the direct effect of the addition of SBW25.

### Comparison of the community diversity metrics

**Figure 5:**
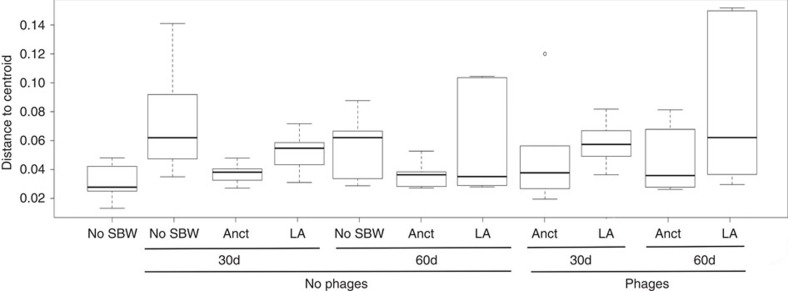
Beta diversity. Box plot representation of the values of the distances to centroid for treatment–time point combinations; No SBW25, ancestral (Anct) and locally adapted (LA) treatments at 30 and 60 days in the absence and presence of phages, based on UniFrac distances. The box plots show medians (horizontal line in box), 25 and 75% quartiles, and max/min values, outliers marked as circles.

**Figure 6:**
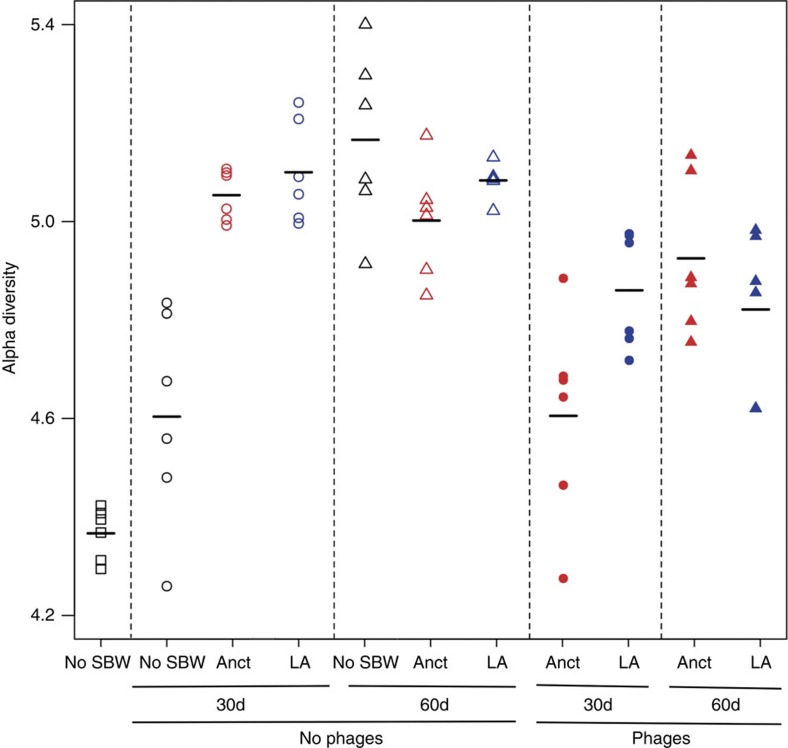
Shannon diversity index. Representation of the within-population diversity values of the community from No SBW25, ancestral and locally adapted treatments at 30 and 60 days in the absence and presence of phages. Square represents day 0, circle represents day 30, triangle represents day 60; black represents No SBW25; red represents ancestral (Anct); blue represents locally adapted (LA); open symbols indicate no phage, closed symbols indicate with phage (+φ).

There was no overall difference in between-community diversity (beta-diversity) across time and treatments (Fig. 5, permutation test; *P*=0.09). Moreover, there was also no overall difference in mean within-community diversity between any treatment (Fig. 6; LMM, *F*_4,26_=1.72, *P*=0.17) or between time points (Fig. 6, LMM, *F*_2,26_=2.11, *P*=0.14).

## Discussion

While contemporaneous evolution of a focal species has been shown to affect the structure of natural communities, its importance relative to purely ecological effects of the presence of the species has not been tested directly. Here, we show that local adaptation of a focal strain of bacteria (*P. fluorescens* SBW25) for a matter of weeks influenced the subsequent structure of a natural compost microbial community as much as the presence of that strain *per se*. Local adaptation, however, had a much smaller relative effect than species presence on community population dynamics, and neither treatments significantly affected community diversity metrics. Moreover, the relative impact of local adaptation was similar regardless of the presence of an SBW25-specific virus that resulted in SBW25 density reductions comparable to the increase in density resulting from local adaptation, suggesting our findings are likely to be robust in the face of additional strong selection pressures.

Although we cannot explain the specific changes in community structure caused by SBW25, general niche theory^23^ would suggest that bacteria that share a similar ecological niche to SBW25 are likely to be negatively affected by its presence and more so if it is locally adapted. Consistent with this view, we found the density of bacteria that are favoured by the same high nutrient culture media on which SBW25 thrives, to be lower in the presence versus absence of SBW25 and lower still when SBW25 had been locally adapted. Moreover, their density was increased when SBW25 density was reduced by the phage. Mechanistically, the production of antimicrobials^24^ is a key trait that may link SBW25 adaptation with altered community composition, since these antimicrobial products play important roles in interactions between soil-associated Pseudomonads and other members of the community^25^.

In contrast to the effects on community composition, it is notable that the effect of local adaptation had relatively little impact on other community characteristics. First, the density of readily culturable bacteria was little affected compared with the effect of the presence of SBW25 *per se*, highlighting the unpredictability of the cascading effects of small changes in the frequency of specific taxa across the whole community. Second, the lack of effect on within- and between-population diversity alongside the clear difference in community composition emphasize the role of deterministic (niched-based) processes, in structuring these compost communities^26^.

The frequency of bacterial OTUs (that is, taxa detected from sequencing) varied between treatments and time points, often in the opposite way. However, one consistent finding across time is that SBW25 (especially when locally adapted) increased the frequency of Firmicutes. We do not know why these particular changes in the community occurred, but these findings are consistent with recent work on mouse gut microbial communities where large increases in the frequency of Firmicutes (the phylum to which the Bacillales belong) resulted from upregulation of secondary metabolites by *Escherichia coli*^27^.

Although our data suggest that the density of a given species is a predictor of its impact on the community as a whole, it is by no means the complete explanation. For example, we found that the presence of phages had a tendency to reduce the impact SBW25 had on the community, but this impact was much less for locally adapted than ancestral SBW25, despite a comparable phage-imposed density reduction. This effect of phages may be because the phages also result in selection for resistance and other correlated traits^20^,^28^,^29^, and these consequences may have differed between ancestral and locally adapted populations. It may also be that ancestral and locally adapted SBW25 interact with other co-occurring species in different ways regardless of the impact of phages. Consistent with this view, the locally adapted SBW25 population had adaptively diversified, with different genotypes having different catabolic profiles that suggest the ability to use different resource^17^. The importance of genotypic diversity on community structure has been highlighted in other systems. For instance, increased genotypic diversity of the perennial plant *Solidago altissima* had a major impact on arthropod species diversity^30^.

Our experiment was specifically designed to compare the effect of adaptive contemporaneous evolution with species presence. Of course, in many contexts, single species will not evolve in isolation, but instead species would be simultaneously (co-)evolving, and it is unclear whether this would increase or decrease the relative importance of contemporaneous evolution in determining community structure. Our results do, however, have clear ecological relevance for colonization following environmental perturbations. Colonization order is a key factor determining community structure because early colonists can reach high densities and dominate resources before other species arrive (‘priority effects')^31^,^32^,^33^,^34^. Our work is consistent with previous theoretical and empirical studies showing that evolution occurs on ecological time scales^3^,^4^,^5^,^6^,^7^,^8^,^9^,^10^,^11^,^12^,^13^,^14^,^15^, and hence, early colonization can further enhance priority effects by allowing species to become locally adapted^35^,^36^,^37^,^38^,^39^. More specifically, we show that priority effects resulting from local adaptation can be equally important as ecological priority effects driven by the mere presence of a species. In summary, contemporaneous evolution is likely to play an important role in the structure and function of natural microbial communities particularly in cases where existing communities are strongly perturbed, including the plant rhizosphere in agricultural systems and the gut microbiome exposed to antibiotics^40^.

## Methods

### Bacterial inoculation into soil-microcosms and sampling

We had previously cultured and frozen six replicates of gentamicin-resistant *P. fluorescens* SBW25 in compost microcosms for 48 days under the same abiotic conditions used for the current experiment^28^. These populations were mixed, grown overnight at 28 °C in King's Media B (KB)^41^ and then ∼10^6^ colony-forming units (CFUs) g^−1^ of soil were inoculated into 12 replicate sterile soil-microcosms. In parallel, 12 microcosms were inoculated with the same density of overnight cultures of the gentamcin-resistant SBW25 ancestor. These soil-microcosms were based on polypropylene trays (10 × 10 cm) containing 100 g of twice-autoclaved compost (Verve Multi-Purpose Compost, UK) soil^18^,^20^. The next day, half the microcosms (six per treatment) were inoculated with 5 ml of M9 salt solution (12.8 g l^−1^ Na_2_HPO_4_-7H_2_O; 3 g l^−1^ KH_2_PO_4_; 0.5 g l^−1^ NaCl; 0.1 g l^−1^ NH_4_Cl) containing a suspension (10^6^ plaque-forming units) of the virulent bacteriophage SBW25φ2 (ref. 19). After 10 days, all microcosms were then inoculated with 3 ml of a soil-wash (20 g of soil compost per 100 ml M9 buffer) containing the resident microbial community (∼10^5^ CFUs g^−1^ soil), an extra six microcosms were also inoculated with soil-wash for the no SBW25 treatment^20^. Soil microcosms were placed in an environmental chamber at 26 °C and 80% relative humidity, conditions fixed to allow a balanced bacterial growth of the whole community. Soil samples (2 g) were collected at 0, 30 and 60 days^18^,^20^,^21^,^42^, and each soil sample was vortexed for 1 min with M9 buffer (5 ml g^−1^ soil). The resultant soil washes were used to determine *P. fluoresncens* and culturable bacteria densities by plating onto KB agar supplemented with gentamicin (15 μg ml^−1^ KB) and LB media, respectively. Note that we used a ‘mark-recapture' approach to follow the ecological and evolutionary dynamics, where there were no culturable bacteria in the compost that were able to grow on gentamicin^18^,^20^. Soil washes were also used to extract total genomic DNA and identify the bacterial community composition by 16S rDNA amplicon sequencing. Note that from each replicate population and time point sampled, a soil-microcosm suspension was stored at −20 °C in glycerol solution (20%).

### Relative fitness assay

The relative fitness of the locally adapted *P. fluorescens* SBW25 population was determined by standard 5-day competition experiments^43^ against a marked strain of ancestral SBW25-*lacZ*^44^ in soil in the presence and absence of the microbial community. Approximately 10^7^ CFUs from both SBW25 strains (overnight cultures grown by shaking at 28 °C) were co-inoculated into 24 sterilized soil-microcosms, half of them containing the resident microbial community. To compare the relative fitness of the locally adapted SBW25 population to the ancestral SBW25-*lacZ* strain under both conditions, here we used the estimation of selection coefficient (*S*), which was calculated by the difference between the estimated Malthusian parameter (*m*)^43^; where *m*=ln(*N*_f_/*N*_0_), and *N*_0_ is the initial and *N*_f_ the final bacteria density of the population after 5 days of competition. Bacterial population densities were determined from plating on LB agar containing X-gal (40 μg ml^−1^).

### DNA isolation and 16S rDNA amplicon library generation

Total genomic DNA was isolated from each replicate population and time point sampled using a FastDNA spin kit for soil (MP Biomedicals, Solon, OH, USA) following the manufacturer's instructions. For the 16S rDNA gene amplicon sequencing, a 254 bp conserved fragment from the V4 hypervariable region was targeted using N501f (5′- AATGATACGGCGACCACCGAGATCTACACTAGATCGCACACTCTTTCCCTACACGACGCTC -3′) and N701r (5′- CAAGCAGAAGACGGCATACGAGATTCGCCTTAGTGACTGGAGTTCAGACGTGTGCTC -3′) primers and with a pool of indexed primers (sequences can be found in Supplementary Data 2) suitable for multiplex sequencing with Illumina technology^45^. After quantification by fluorometric, 5 ng genomic DNA was mixed with 0.25 μl of each 16S primer (10 μM) and 0.5 μl of each of the nested primers (10 μM). KAPA amplification mix (2 × ) was used and the final volume was 20 μl. A negative control of water eluted from the FastDNA spin kit was also included. The samples were amplified at the following conditions: 98 °C for 2 s (one cycle), 95 °C for 20 s, 65 °C for 15 s, 72 °C for 30 s (25 cycles), 72 °C for 5 min (one cycle), 4 °C hold. The samples were then cleaned up using Agencourt Ampure XP beads (Beckman Coulter) at a ratio 1:1. The products were eluted in 12 μl 10 mM Tris pH 7.5. The samples were analysed by Qubit fluorometry and Bioanalyser. For each protocol, a total of 42 MiSeq sequence libraries were made from seven groups, with six replicates per group.

### Sequencing and bioinformatic analysis

Amplicon sequencing was performed by Illumina MiSeq technology at Centre for Genomic Research (University of Liverpool). Each pool of amplicons was sequenced at 2 × 250 bp paired-end sequencing with chemisty v2. Sequence data were performed using an adjusted pipeline; Casava v1.8.2 and Cutadapt v1.2.2 were used to perform the basecalling, de-multiplexing and trimming of the indexed reads^22^,^46^,^47^. Per sample, 54,000–260,000 filtered read pairs were analysed, and assembled into a single sequence by Flash^48^, and then Qiime v1.8 was used for metagenomic analysis^22^. Clustering sequences at 97% of similarity generated 1,298 OTUs, the *de novo* OTU-picking and their quantification was done by using USEARCH^49^ v7.0. Sequences falling below the 97% similarity threshold for any of the OTUs clusters were removed from further analyses, to act as a filter against potentail artefacts caused by sequencing error. The Greengenes database of ribosomal RNA sequences^50^ v12.8 was used as reference for chimera detection and taxonomy assignments. The taxonomic assignments for each OTU was performed by using Qiime v1.8 and RDP classifier^51^.

### Resequencing methods

DNA was extracted from one randomly picked clone from each of the six locally adapted replicas of gentamicin-resistant *P. fluorescens* SBW25 and a clone of ancestral SBW25. Illumina TruSeq libraries were prepared and paired-end sequence generated on an Illumina GAIIx platform. Reads were mapped to the SBW25 reference genome (GenBank NC_012660.1) using bwa (v0.5.9-r16), with local realignment and variant calling (relative to the ancestral SBW25 genome sequenced at the same time) achieved using GATK UnifiedGenotyper (v2.1-13-g1706365) followed by snpEff (v4.1) to assign effects on coding genes.

### Statistical analyses

The impact of the different treatments on bacteria densities and within-population (alpha) diversity (Shannon index) during the 60-day experiment was determined using Linear Mixed Effects Models, where density was log-transformed, and treatments and time fitted as fixed effects, and population fitted as a random factor. A one-sample *t*-test was used to determine whether the relative fitness for ancestral and locally adapted SBW25 differed from each other. These analyses were carried out using JMP software v9. The phyloseq and vegan packages in R were used to calculate alpha- and beta-diversity and perform analyses and to produce two-dimensional Principal Coordinates Analysis plots using weighted UniFrac^52^ distances from the relative abundances of taxa for each sample. For beta-diversity analysis, the betadisper function in the vegan package was used to test for multivariate homogeneity of group dispersions using a permutational approach^53^. PERMANOVA (in R) was used to determine differences (based on weighted UniFrac distances) being treatments and time points. Bootstrapped 95% CIs for pairwise differences between groups were calculated in R, to allow comparison of magnitude of these pairwise differences.

### Data availability

All sequencing information and data that support the findings of this study have been deposited in the European Nucleotide Archive (ENA) database with the accession codes PRJEB9900 and PRJEB13609. The authors declare that all other data are contained within the article and its Supplementary Files.

## Additional information

**How to cite this article:** Gómez, P. *et al*. Local adaptation of a bacterium is as important as its presence in structuring a natural microbial community. *Nat. Commun.* 7:12453 doi: 10.1038/ncomms12453 (2016).

## Supplementary Material

Supplementary Data 1 List of the SNPs and INDELs present in each of the six locally adapted *P. fluorescens* SBW25 clone.

Supplementary Data 2 List of index adapter primer sequences used for each sample.
